# The factors that limit activities of certified diabetes educators in Japan: a questionnaire survey

**DOI:** 10.1186/2193-1801-3-611

**Published:** 2014-10-17

**Authors:** Miyako Kishimoto, Mitsuhiko Noda

**Affiliations:** Department of Diabetes, Endocrinology, and Metabolism, Center Hospital, National Center for Global Health and Medicine, 1-21-1 Toyama, Shinjuku-ku, Tokyo, 162-8655 Japan; Diabetes and Metabolism Information Center, Diabetes Research Center, Research Institute, National Center for Global Health and Medicine, 1-21-1 Toyama, Shinjuku-ku, Tokyo, 162-8655 Japan; Department of Diabetes Research, Diabetes Research Center, Research Institute, National Center for Global Health and Medicine, 1-21-1 Toyama, Shinjuku-ku, Tokyo, 162-8655 Japan

**Keywords:** Certified diabetes educators, Japan, Job satisfaction, Self-perception

## Abstract

**Background:**

The certified diabetes educator (CDE) is a qualification awarded to health professionals with specialized knowledge, skills, and experiences in diabetes management and education. To clarify whether CDEs consider themselves to be working sufficiently, in other words, making sufficient use of their specialized skills or not, a questionnaire survey was conducted.

The participants were persons involved in diabetes-related educational seminars and medical personnel engaged in diabetes care at the National Center for Global Health and Medicine. They were asked to complete a questionnaire regarding self -perception of CDE’s activities and to describe the reasons for their answers.

**Findings:**

Fewer than 40% of the responding CDEs in each of the professions surveyed were satisfied with the current state of their activities and contributions as a CDE. For CDEs, “lack of labor” is the most concerning issue that limits their satisfactory activities as CDEs, followed by “condition of facilities”. Other factors such as insufficient “interprofessional teamwork”, “limited personal ability”, “mismatched allocation”, and “low recognition for CDEs” also limited their activities.

**Conclusion:**

Many CDEs perceived they are not working sufficiently. Further efforts should be made to support CDEs to improve their working conditions.

## Introduction

Globally, the number of patients with diabetes has been increasing rapidly and become a significant health care burden to each country. As a result of the complex nature of this disease process, patients with diabetes require comprehensive management and support. However, limited numbers of physicians alone cannot cope with the significant increase in the number of diabetic patients in recent years. Therefore, developing health care professionals who apply detailed knowledge and skills in diabetes care and can be members of interprofessional teams, such as certified diabetes educators (CDEs), is a pressing need. The certified diabetes educator of Japan (CDEJ) is a qualification awarded to a nurse, dietitian, pharmacist, clinical laboratory technician, or physical therapist possessing wide specialist knowledge on diabetes. CDEJs have passed the required examinations and been approved by the Japanese Certification Board for Diabetes Educators established in 2000 (Kawaguchi 2007). Currently, there are nearly 18,400 CDEJs, mostly nurses (47.8%), dietitians (23.6%), and pharmacists (15.0%) (Certification Board for Diabetes Educators in Japan 2014). In addition, there are CDEs who play active roles in local areas, called local certified diabetes educators (LCDE). LCDEs are developed and qualified in a manner befitting the conditions of an individual local area. Compared to CDEJ, the LCDE qualification has less stringent eligibility requirements for examination and a wide range of medical professions can apply for it; therefore, the number of LCDEs has been increasing and contributing to improvement in diabetes care in local areas. Although incorporating diabetes educators into clinical services is expected to improve clinical and quality of life outcomes for persons with diabetes (Burke et al. 2014) and many healthcare professionals recognize the need for more diabetes educators such as diabetes specialist nurses (Peyrot et al. 2013), levels of perception and satisfaction of CDEs themselves for their activities are unclear and often unexpressed. To clarify these points, a questionnaire survey was conducted.

## Methods

The National Center for Global Health and Medicine (NCGM) in Japan has been conducting free-for-all styled educational seminars five to six times per year for more than five years for various professional groups who are actually involved and interested in diabetes care (Kishimoto and Noda 2013). The participants of the NCGM educational seminars and other related seminars, as well as NCGM medical staff who are engaged in diabetes care, were asked to report their profession, whether they were CDEs or not, and to complete a questionnaire featuring 3 questions. Question 1 (Q1) was for those who were CDE, “do you think that you are working sufficiently as a CDE”, which required a “yes”, “no”, or “not sure” response. Question 2 (Q2) was “why do you think so? Please explain the reasons for this”. Question 3 (Q3) was “please state your ideas regarding how to resolve this problem”. Q2 and Q3 were answered by free description. The questionnaire was anonymous and the participants’ responses to the questionnaire were voluntary. The answers for Q1 were analyzed by response percentages of each profession. Those who answered “no” and “not sure” to Q1 were further analyzed in combination with the answers for Q2. Substantially similar answers for Q2 were grouped and categorized into themes.

## Results

Of 744 people offered the questionnaire (725 seminar participants and 19 NCGM medical staff), 498 people answered it (respondents). The number and the percentages of each occupation in the respondents were as follows: physicians, 29 (5.8%); nurses, 167 (33.5%); pharmacists, 157 (31.5%); registered dieticians, 98 (19.7%); clinical laboratory technicians, 31 (6.2%); physical therapists, 7 (1.4%); and others including students or dental hygienists, 9 (1.8%). The percentages of CDE acquisition in each occupation eligible to apply for CDE were as follows: nurses 56.9%, pharmacists 17.2%, registered dieticians 61.2%, clinical laboratory technicians 58.1%, and physical therapists 71.4%. The number and percentages of responses to Q1 are shown in Table 1. Fewer than 40% of the CDEs in each of the professions surveyed were satisfied regarding the current state of their activities and contributions as a CDE. Representative answers to Q2 and Q3 for CDEs in each profession are shown in Table 2. The answers for Q2 were varied. Those who answered “no” and “not sure” to Q1 were classified into six thematic categories. Answers that considered the fundamental obstacle to CDE’s activity to be busy medical staff who devoted so much to their own professional daily duties that they could not participate in any additional work as CDEs were categorized under “lack of labor”. Answers such as “Because there are no expert physicians in diabetes in the current working place and patients with diabetes are few” or “The facility does not seems to put an effort to diabetes and does not expect CDE to contribute” or “because of limited health insurance reimbursement, some CDE services are unwelcome to the facility” were categorized under “condition of facilities”. Answers that considered lack of “interprofessional teamwork” as an obstacle to CDE’s activities included "interprofessional communication is insufficient and cannot share patient’s information” or “other medical staff are not cooperative to CDEs”. Answers that considered lack of knowledge or experience as the main reason CDEs feel that they are not working sufficiently were categorized as “limited personnel ability”. Answers such as “I’m working at an operating room and have no chance to work as CDE” or “I am allocated to the ward where there are no diabetic patients” viewed job allocation as the main problem and were categorized in “mismatched allocation”. Finally, answers that suggested poor recognition of CDEs by other medical staff or patients led to limited requests for CDE’s activities were categorized as “low recognition for CDE”. The percentages of each occupations’ answers classified into the aforementioned six thematic categories are shown in Table 3. For CDEs who were nurses, pharmacists, registered dieticians, and clinical laboratory technicians, “lack of labor” was the most concerning obstacle limiting their satisfactory activities as CDEs, followed by “condition of facilities”.

**Table 1:** **Answers by profession to Question 1**

Profession	Yes	No	Not sure	Non-response
Nurse	12 (12.6%)	53 (55.8%)	27 (28.4%)	3 (3.2%)
Pharmacist	3 (11.1%)	10 (37.0%)	14 (51.9%)	0 (0.0%)
Registered dietician	13 (21.7%)	23 (38.3%)	23 (38.3%)	1 (1.7%)
Clinical laboratory technician	6 (33.3%)	7 (38.9%)	5 (27.8%)	0 (0.0%)
Physical therapist	2 (40.0%)	3 (60.0%)	0 (0.0%)	0 (0.0%)

Each column indicates numbers and percentages of respondents in each category.

**Table 2:** **Representative descriptions of CDEs by profession for responses to question 2 and proposals for improvement**

Profession	Explanations
Nurses	**Reasons for “Yes”**
	➢ I am educating diabetic patients with other CDEs
	➢ Physicians are cooperative with us.
	➢ Interdisciplinary teamwork works well
	➢ I feel that I can manage daily consultation for patients.
	**Reasons for “No”**
	➢ Too busy to spare my time for CDE’s service. We are required to prioritize our own section’s work and cannot have enough time to talk with diabetic patients.
	➢ The physician who I am working with hates to listen to my comments.
	➢ Interprofessional communication is insufficient and I cannot expect cooperation from other professions.
	➢ I was allocated to the section I can hardly see diabetic patients.
	➢ I do not think that CDEs are well recognized.
	**Reasons for “Not sure”**
	➢ Each CDE works individually and I think interprofessional teamwork is insufficient.
	➢ I am the only CDE in my hospital and what I can do for patients is limited.
	➢ I’m instructing insulin therapy for outpatients, but I’m not sure whether physicians and patients assess my work.
	**Proposal for improvement**
	➢ We need more staff so that CDEs can have more time to provide their special service to patients.
	➢ Establish interprofessional teamwork among CDEs so that CDEs can appeal their activities more efficiently and can increase the recognition for their existence.
	➢ Not just complaining that we are not recognized, we ourselves should take an action to advertise CDE’s ability and should let others know more about CDE’s service that can get health insurance reimbursement.
	➢ We ourselves should improve our knowledge and abilities.
	➢ Not just waiting other people ask us to do something; CDEs themselves should plan a project and submit it to the facility.
Pharmacist	**Reasons for “Yes”**
	➢ Lectures to the local residents and educational classes for patients are being held and CDEs are involved.
	**Reasons for “No”**
	➢ Too busy doing other duties that I cannot provide a CDE’s service.
	➢ There is no diabetologist in my hospital. Without physicians’ cooperation, we cannot manage diabetes efficiently.
	➢ I was allocated to an irrelevant section so that I cannot contribute as a CDE.
	➢ I’m the only CDE in my hospital. Now I’m recommending a nurse become a CDE so that we can work together.
	**Reasons for “Not sure”**
	➢ I’m attending interprofessional meetings and educational classes for patients, but I don’t think that is sufficient.
	➢ Not only in my field, I think I’m able to educate patients using my knowledge about diet and exercise therapy. However, other pharmacists are not interested and I’m afraid I cannot continue my service as a CDE.
	➢ CDEs are not sufficiently recognized. Even though educational classes for patients are operated, the hospital does not evaluate them.
	➢ There are few diabetic patients in my hospital.
	**Proposals for improvement**
	➢ Each CDE is working individually; however, for efficient diabetes management, interprofessional teamwork will be necessary.
	➢ Satisfy enough number of medical staff so that CDEs can have more time to spare their special activities.
	➢ CDEs should develop their skill and knowledge and advertise their ability to other medical staff.
Registered dietician	**Reasons for “Yes”**
	➢ After I became a CDE, I think I can answer questions from patients smoothly with self-confidence.
	➢ I think I have been working effectively as a member of an interprofessional team.
	➢ I have been contributing to educating patients through daily counseling and educational classes.
	**Reasons for “No”**
	➢ Owing to the lack of labor, I cannot work sufficiently as a CDE.
	➢ It is impossible to work only for diabetic patients in my situation. I feel that I just have CDE qualification but cannot use it effectively.
	➢ I am a leader of a nutrition support team at the university attached hospital, and don’t have time for additional CDE service.
	➢ Hard to cooperate and share patient’s information with other professionals who are not CDE.
	➢ I feel that what I do as a dietician now is not different from what other non-CDE dieticians do.
	➢ Other medical staff do not think CDE is necessary
	**Reasons for “Not sure”**
	➢ There are only two CDEs in my hospital, a nurse and myself, and I cannot say that interprofessional teamwork works well. However, I feel that I have been contributing to improve diabetes management of my hospital.
	➢ Although, we are organizing an association for patients with type 2 diabetes and also for children with type 1 diabetes, I still think that there is something more that I can do as a CDE.
	**Proposals for improvement**
	➢ Each staff should develop his or her own knowledge and experiences to maintain their ability as CDEs and to win recognition.
	➢ Positive action will be necessary to establish interprofessional networks both inside and outside of hospital. That will make a favorable outcome in diabetes care and make others recognize the importance of CDEs.
Clinical laboratory technician	**Reasons for “Yes”**
	➢ There are CDEs in the four professions in my hospital and regular interprofessional meetings are conducted. Each staff fills in their comments about patient’s education in one record so that we can share the information.
	➢ I’m working at a small clinic and have time to talk with patients while I’m collecting their blood samples. I try to use my knowledge and experience as a CDE and do my best to answer patient’s questions.
	**Reasons for “No”**
	➢ Due to the nature of my profession, compared to other staff, I think I have less opportunity to talk with patients.
	➢ I can’t spare time for CDE service because that will increase burden on other staff in my section.
	➢ I have been educating patients my own way. I should have learned more.
	**Reasons for “Not sure”**
	➢ Interprofessional meeting and educational classes for patients have been held but I don’t think it is enough. However, we are too busy and cannot do more.
	➢ I have time to talk with patients but not enough to do profound education.
	**Proposal for improvement**
	➢ Tighten and increase the amount of interprofessional communication among CDEs.
Physiotherapist	**Reasons for “Yes”**
	➢ CDEs play a central role and made a diabetes care team. Educational classes for patients are held once a week and a walk rally is organized once a year.
	➢ In addition to activities inside the hospital, CDEs give lectures to the residents in our areas.
	**Reasons for “No”**
	➢ Interest and motivation for diabetes care is low in my hospital.
	➢ Interprofessional teamwork for diabetes care is insufficient.
	**Proposal for improvement**
	➢ We should show certain data demonstrating the importance and necessity of CDEs.

**Table 3:** **Thematic analysis for CDEs**

	Nurse	Pharmacist	Registered dietician	Clinical laboratory technician	Physical therapist
Lack of labor	23 (28.8%)	12 (44.4%)	8 (17.4%)	3 (25.0%)	0 (0.0%)
Condition of facilities	12 (15.0%)	6 (22.2%)	8 (17.4%)	1 (8.3%)	1 (20.0%)
Interprofessional teamwork.	10 (12.5%)	6 (22.2%)	7 (15.2%)	1 (8.3%)	1 (20.0%)
Limited personal ability	4 (5.0%)	0 (0.0%)	3 (6.5%)	2 (16.7%)	0 (0.0%)
Mismatched allocation	7 (8.8%)	4 (14.8%)	2 (4.3%)	0 (0.0%)	0 (0.0%)
Low recognition for CDE	4 (5.0%)	2 (7.4%)	2 (4.3%)	0 (0.0%)	1(20.0%)

Each column indicates numbers and percentages of respondents in each category.

## Discussion

The present questionnaire survey indicated that many CDEs surveyed considered that CDEs themselves were not working sufficiently as CDEs. Previous research has shown that successful intervention by CDEs helped to improve clinical and quality of life outcomes in certain patient groups (Burke et al. 2014; Anderson and Funnell 2008; Kahn et al. 2012; Barlow et al. 2005). However, CDEs’ activities with diabetes care have certain difficulties in clinical settings. In the present study, reasons that limited CDE’s activities were varied and differed by professions; however, “lack of labor” was the main problem followed by “conditions of facilities”. These two problems may be difficult to solve solely by CDEs.

Other factors such as insufficient “interprofessional teamwork” can be improved by continuous efforts by members. Interprofessional teamwork in diabetes care is extremely important (Dounis et al. 2014; Ritholz et al. 2011; Kishimoto and Noda 2014) and the successful use of CDEs on interprofessional teams suggests that expansion of the team approach might be effective in the provision of diabetes education services (Langelier et al. 2010) and may raise CDE recognition levels. The factor of “limited personal ability” can be improved by individual effort and may lead to correcting mismatched allocations.

Langelier et al. summarized the findings of a statewide survey of CDEs in New York and reported several factors that contributed to limited demand for their services. For example, “limited health insurance reimbursement for CDE services” interfered with their ability to serve patients in need of diabetes education as well as “lack of timely referrals” and “lack of physician and patient awareness of the competencies of CDEs”. Langelier et al. also suggested that physicians could be educated on the value of using CDEs in the delivery of diabetes education services, and reimbursement for the provision of diabetes education services could be increased and expanded to cover services that improve outcome for patients with diabetes. The procedures of CDE acquisition and the range of authorized services done by CDEs may vary in each area and country. However, their proposal can be adopted in Japan.

In addition to the answers to the questions, some respondents provided additional comments regarding difficulties in acquisition and renewal of CDE qualification. To become a CDEJ, medical personnel in mentioned professions need to work continuously for at least two years in medical institutions, have diabetes education experience for at least 1,000 h, participate in two day workshops organized by the Certification Board, submit a report of 10 cases of diabetes education they have conducted, and pass the required examinations. To apply for renewal of certification every five years, CDEs must participate in scientific meetings and trainings to obtain re-certification credits (Kawaguchi 2007). Some CDEs and CDE candidates consider that meeting certification requirements are too strict and can be costly and time-consuming. Therefore, some CDEs have given up qualification renewal. To supply enough CDEs in clinical settings, reconsideration of the system of CDE acquisition and renewal will be necessary. In addition, providing higher salaries to CDEs could be one of the strategies to increase CDEs; employers can demonstrate that they value the certification and special knowledge of CDEs by paying more (Langelier et al. 2010).

There are some limitations in the present survey. First, because of the nature of the term, the meaning of “working sufficiently” may vary between individuals and respondents might have difficulties in answering questions. This might be one of the reasons why many respondents answered “not sure” to Q1 and Q2, instead of giving more definite answers, such as “yes” or “no”. Second, because the questionnaire was conducted for the participants of educational diabetes seminars who might be more enthusiastic and highly motivated in diabetes care than persons who did not attend the seminars, the results might be biased and unrepresentative in general. Third, because of too few clinical laboratory technicians and physical therapists, our findings may not be representative in these professions.

## Conclusion

In conclusion, the results of the questionnaire survey demonstrate that many CDEs consider they are not working sufficiently. As CDEs are an important part of diabetes care, we should recognize the factors that obstruct CDE’s activities and should make further efforts to support them to improve their working conditions.
